# Comparative Transcriptome Analysis Revealed Candidate Gene Modules Involved in Salt Stress Response in Sweet Basil and Overexpression of *ObWRKY16* and *ObPAL2* Enhanced Salt Tolerance of Transgenic *Arabidopsis*

**DOI:** 10.3390/plants13111487

**Published:** 2024-05-28

**Authors:** Yukun Wang, Hong Ye, Fei Ren, Xiaoqiang Ren, Yunna Zhu, Yanhui Xiao, Jinming He, Bin Wang

**Affiliations:** 1 Guangdong Provincial Key Laboratory of Utilization and Conservation of Food and Medicinal Resources in Northern Region, Shaoguan University, Shaoguan 512005, China; wangyu_kun1@163.com (Y.W.); zhuyn326@126.com (Y.Z.); yhxiao@sgu.edu.cn (Y.X.); 2 College of Biology and Agriculture, Shaoguan University, Shaoguan 512005, China; 19881212hong@163.com (H.Y.); ren_faye_sgu@163.com (F.R.); rxq_81@126.com (X.R.); 3 Engineering and Technology Research Center of Shaoguan Horticulture in Shaoguan University, Shaoguan 512005, China

**Keywords:** transcriptome, WRKY, salt stress, phenylalanine ammonia lyase, sweet basil, transgenic *Arabidopsis*

## Abstract

Sweet basil (*Ocimum basilicum* L.) is an important aromatic plant with high edibility and economic value, widely distributed in many regions of the tropics including the south of China. In recent years, environmental problems, especially soil salinization, have seriously restricted the planting and spread of sweet basil. However, the molecular mechanism of the salt stress response in sweet basil is still largely unknown. In this study, seed germination, seedling growth, and chlorophyll synthesis in sweet basil were inhibited under salt stress conditions. Through comparative transcriptome analysis, the gene modules involved in the metabolic processes, oxidative response, phytohormone signaling, cytoskeleton, and photosynthesis were screened out. In addition, the landscape of transcription factors during salt treatment in sweet basil was displayed as well. Moreover, the overexpression of the WRKY transcription factor-encoding gene, *ObWRKY16*, and the phenylalanine ammonia-lyase-encoding gene, *ObPAL2*, enhanced the seed germination, seedling growth, and survival rate, respectively, of transgenic *Arabidopsis*, suggesting that they might be important candidates for the creation of salt-tolerant sweet basil cultivars. Our data enrich the study on salt responses in sweet basil and provide essential gene resources for genetic improvements in sweet basil in the future.

## 1. Introduction

Soil salinization is one of the major environmental stresses faced by plants. Soil salinization seriously restricts growth and development and significantly affects plant yield [1]. One-third of the world’s agricultural land is under threat from salt stress [2]. Plants can be divided into halophytic and glycophytic plants according to their salt tolerance characteristics. Most crops are glycophytic plants, which are sensitive to high concentrations of salt in the soil. Many studies indicate that unreasonable applications of chemical fertilizer, excessive irrigation, and industrial pollution are the main causes of soil salinization [3]. Salt stress is usually caused by high concentrations of sodium ions (Na^+^) and chloride ions (Cl^−^) in the soil [4]. There are three types of high salt stress: ionic stress, osmotic stress, and secondary stress [1]. Ionic stress causes harmful effects on plant growth and development through the toxic effect of salt ions on cells. Saline ions enter the plant through root absorption, are further transported to the stem over long distances, and eventually accumulate in large quantities in the leaves [5]. High concentrations of Na^+^ in the cytoplasm disrupt the absorption of other ions in plant cells. Since many ions are necessary for the activation and maintenance of enzyme activity, high concentrations of Na^+^ result in harmful effects on many metabolic pathways [1,6,7]. Osmotic stress is caused by high concentrations of salt in soil and water. Excess soluble salt in the soil reduces water uptake by roots, resulting in a water shortage in the plant [8]. Further, osmotic and ionic stress cause secondary plant stress, such as the accumulation of toxic compounds (e.g., hydroxyl radicals, hydrogen peroxide, and superoxide anions) and the disruption of the nutritional balance of plants [1]. However, the molecular mechanism of osmotic stress response in Ocimum plants is still largely unclear.

Sweet basil is an annual herbaceous plant in the Lamiaceae family and is cultivated in subtropical regions worldwide. Sweet basil is used as a culinary herb and has various beneficial properties, including antioxidant and antimicrobial effects. In addition, sweet basil is rich in essential oils and has significant applications in medicine and healthcare. However, as a non-halophytic plant, sweet basil cannot grow well in saline soils, which severely limits its cultivation and spread. So far, research on sweet basil’s response to salt stress is limited, with most studies focused on the impacts of exogenous substances on salt tolerance [9,10] or the changes in phytochemical composition under salt stress conditions [11] in sweet basil. Thus, it is necessary to uncover the molecular mechanism of the salt stress response in sweet basil via omics, molecular, and genetic methods.

Comparative transcriptome analysis is a powerful omics method that can indicate the alterations in gene expression among different samples, resulting in the exposure of gene functions. In this study, comparative transcriptome analysis was performed to elucidate the dynamic changes in the transcriptional level in sweet basil under salt stress. The gene modules in classical and unique GO terms and KEGG pathways were identified as candidate genes involved in the response to salt stress in sweet basil. In addition, the transgenes of *ObWRKY16* and *ObPAL2* in *Arabidopsis* indicated they are essential candidates for the enhancement of salt tolerance in sweet basil. Taken together, the data from this study provides useful gene resources for the molecular breeding and creation of salt-tolerant varieties of sweet basil.

## 2. Results

### 2.1. Effects of Salt Treatment on Growth of Sweet Basil

**Figure 1:**
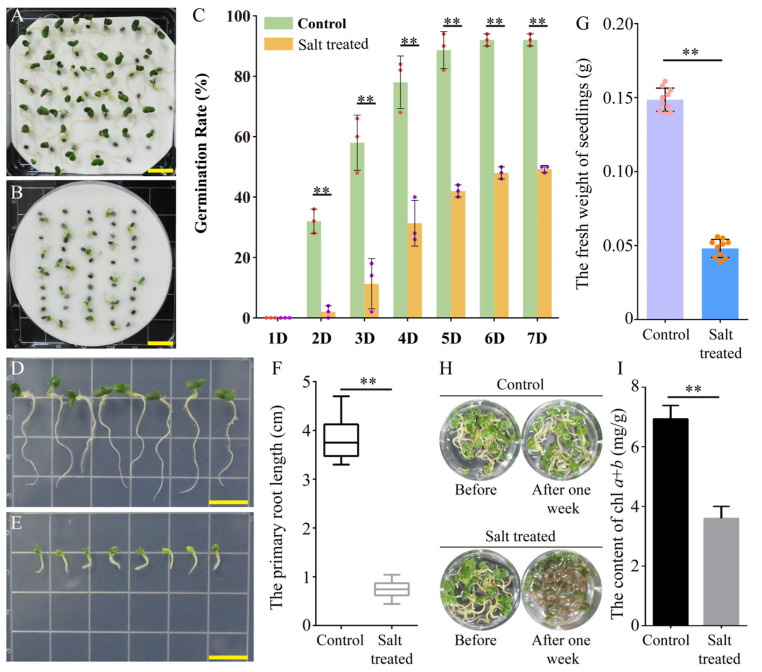
Morphological and physiological changes in sweet basil under salt stress conditions. (**A**) Observation of seed germination in sweet basil under normal conditions. (**B**) Observation of seed germination in sweet basil under salt stress conditions. (**C**) Seed germination rates under normal and salt stress conditions. **: *p* < 0.01. Dots indicated data points. (**D**) Observation of primary root growth in sweet basil under normal conditions. (**E**) Observation of primary root growth in sweet basil under salt stress conditions. (**F**) Quantification of primary root length in sweet basil under normal and salt stress conditions. **: *p* < 0.01. (**G**) The fresh weight of sweet basil seedlings under normal and salt stress conditions. **: *p* < 0.01. Dots indicated data points. (**H**) Changes in sweet basil seedlings after normal and salt treatments. (**I**) Quantification of chlorophyll *a*+*b* content in sweet basil under normal and salt conditions. **: *p* < 0.01.

To understand the growth features of sweet basil under salt stress conditions, we used 150 mM of NaCl solution (salt-treated group) and water (control) to treat seeds and seedlings, respectively. Under the water treatment, the seeds germinated normally, and over 90% of them germinated after one week (Figure 1A,C). For the salt-treated seeds, only 50% of the seeds germinated after 7 days of treatment (Figure 1B,C). Seed germination was inhibited significantly in the salt-treated group compared to the control (Figure 1C). We also measured the primary root lengths of the 10-day-old seedlings with the water and salt treatments. As shown in Figure 1D,E, the growth status of the primary roots in the control was superior to that of the salt-treated group. The quantized data showed the primary roots of the control seedlings were over 3-fold longer than those of the salt-treated seedlings (Figure 1F). Meanwhile, the fresh weight of the control seedlings was significantly higher than that of salt-treated seedlings (Figure 1G). In addition, we compared the changes in chlorophyll contents in the control and salt-treated seedlings. After one week, the color of the roots and cotyledons became dark in the salt-treated seedlings compared with the control seedlings (Figure 1H). Accordingly, the content of chlorophyll *a* + *b* was clearly lower in the salt-treated seedlings than in the control seedlings (Figure 1I). These results suggest that the sweet basil was sensitive to the 150 mM NaCl treatment, and its growth was negatively influenced by salt stress.

### 2.2. Comparative Transcriptome Analysis of Water- and Salt-Treated Samples

**Figure 2:**
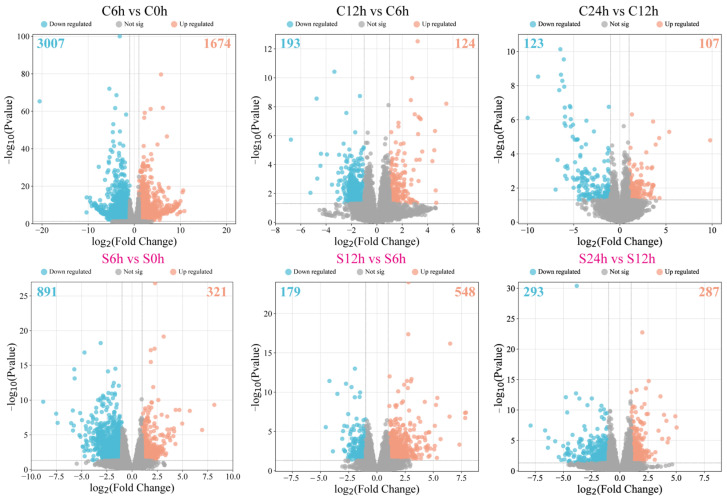
Volcano plots show the DEGs of different comparative groups under normal (C) and salt stress (S) conditions.

In order to understand the dynamic changes in genes at the transcriptional level, we carried out a comparative transcriptome analysis by using samples collected at the 0 h (before treatment), 6 h, 12 h, and 24 h time points. In the water-treated groups, there were 4681 (3007 up- and 1674 downregulated) differentially expressed genes (DEGs) in comparative group C6 h vs. C0 h. In comparative group C12 h vs. C6 h, 193 down- and 124 upregulated DEGs were isolated. A total of 230 DEGs, including 107 upregulated and 123 downregulated genes, were screened out in comparative group C24 h vs. C12 h. In the salt-treated samples, 321 up- and 891 downregulated DEGs were identified in comparative group S6 h vs. S0 h, respectively, and 727 DEGs, including 548 up- and 179 downregulated genes, were detected in comparative group S12 h vs. S6 h. In comparative group S24 h vs. S12 h, 287 up- and 293 downregulated DEGs were confirmed (Figure 2). Based on these results, we found that more DEGs were identified in group C6 h vs. C0 h than in group S6 h vs. S0 h, indicating that salt treatment might inhibit the expression of several genes. However, more DEGs were detected in groups S12 h vs. S6 h and S24 h vs. S12 h than C12 h vs. C6 h and C24 h vs. C12 h, suggesting that multiple salt stress response genes were activated in the sweet basil.

### 2.3. GO Enrichments of Salt-Response Gene Modules

**Figure 3:**
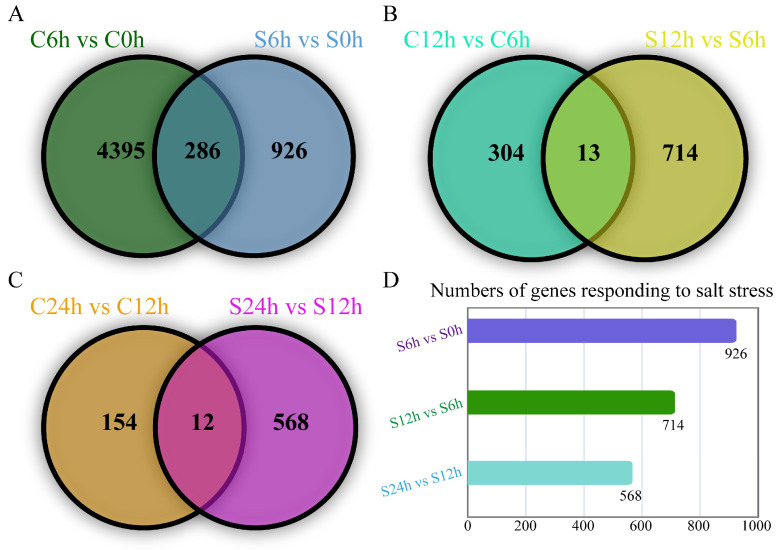
Identification of salt stress response genes. Venn diagrams show the gene numbers in the (**A**) 6 h vs. 0 h group, (**B**) 12 h vs. 6 h group, and (**C**) 24 h vs. 12 h group. The number of salt stress genes in each group is shown in (**D**).

Given many DEGs not only responded to water treatment but also showed altered expression patterns under salt stress, we then found these genes and removed them, finally gaining the unique salt stress response DEGs. We counted the numbers of DEGs in comparative groups S6 h vs. S0 h, S12 h vs. S6 h, and S24 h vs. S12 h, and isolated 926, 714, and 568 salt stress response DEGs, respectively (Figure 3).

**Figure 4:**
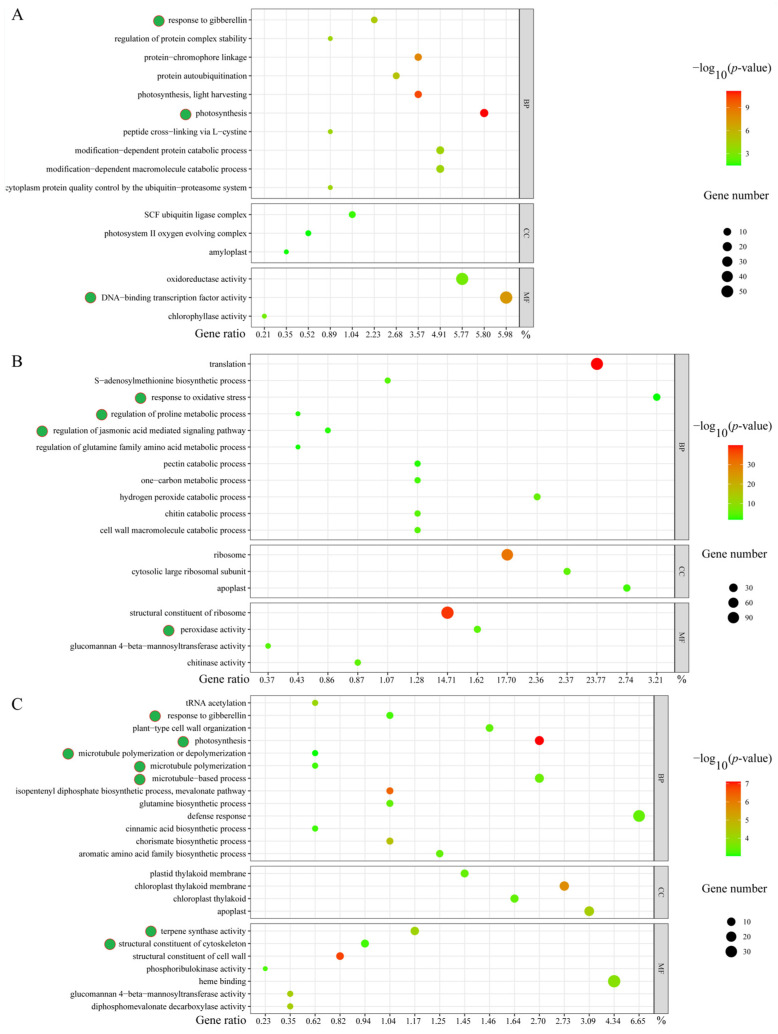
GO enrichment analysis of DEGs in the (**A**) 6 h vs. 0 h group, (**B**) 12 h vs. 6 h group, and (**C**) 24 h vs. 12 h group. Green dots indicate the candidate GO terms.

In order to uncover the salt stress-related gene modules, we performed a Gene Ontology (GO) analysis among the salt stress response DEGs. In comparative group S6 h vs. S0 h, many DEGs were mainly clustered into ‘response to gibberellin’, ‘photosynthesis’, ‘oxidoreductase activity’, and ‘DNA-binding transcription factor activity’ terms (Figure 4A). In comparative group S12 h vs. S6 h, we found several DEGs were enriched into ‘response to oxidative stress’, ‘regulation of proline metabolic process’, ‘regulation of jasmonic acid (JA)-mediated signaling pathway’, and ‘peroxidase activity’ terms (Figure 4B). For the DEGs in comparative group S24 h vs. S12 h, many were linked to ‘response to gibberellin’, ‘photosynthesis’, ‘microtubule polymerization or depolymerization’, ‘microtubule-based process’, ‘terpene synthase activity’, and ‘structural constituent of cytoskeleton’ terms (Figure 4C). These results suggest that gene modules involved in hormone (jasmonic acid and gibberellin) signaling pathways, oxidative stress responses, cytoskeleton stabilities (microtubule structure), and photosynthesis might play important roles in regulating salt stress responses in sweet basil.

**Figure 5:**
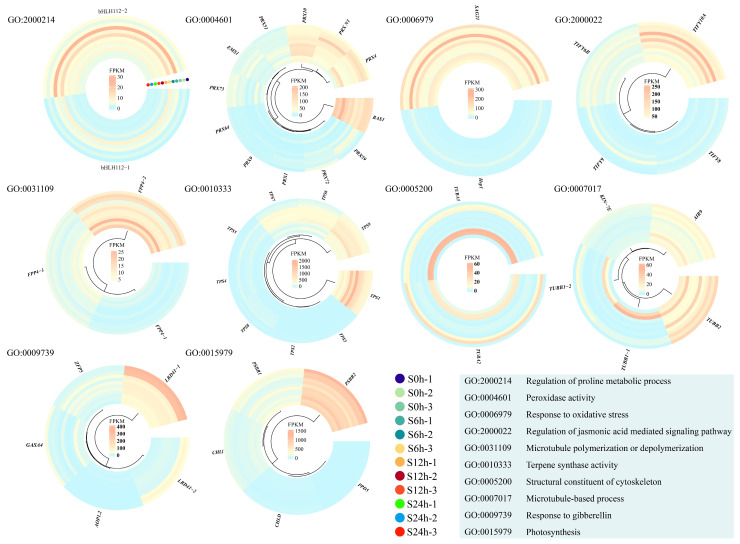
Heatmap of candidate genes isolated in GO enrichment analysis.

Moreover, we built a heatmap of each gene module mentioned above using the FPKM (fragments per kilobase of exon model per million mapped) values to display the expression profiles in the salt-treated samples at 0 h, 6 h, 12 h, and 24 h. There were two gene copies annotated as *bHLH112* in the GO term ‘regulation of proline metabolic process’, and we found that *ObbHLH112-2* showed clear expression changes during salt stress (Figure 5; Table S1). A total of 12 DEGs, including 10 peroxidase-encoding genes (*PRXs*), one leucine-rich repeat receptor protein kinase-encoding gene (*EMS1*), and one 2-Cys-peroxiredoxin-encoding gene (*BAS1*), were linked to the GO term ‘peroxidase activity’. We found three *PRX* genes, *ObPRX4*, *ObPRX10*, and *ObPRX73*, which exhibited distinct expression alterations under salt stress conditions (Figure 5; Table S1). *SENESCENCE-ASSOCIATED GENE 21* (*SAG21*) and *HEAT SHOCK PROTEIN 1* (*HSP1*) were linked to the GO term ‘response to oxidative stress’, and *ObSAG21* had higher expression levels than those of *ObHSP1* (Figure 5; Table S1). Four TIFY genes participated in the JA-mediated signaling pathway, and ObTIFY6B and ObTIFY10A showed obvious expression changes under salt stress (Figure 5; Table S1). A total of 10 genes were linked to cytoskeleton-related GO terms; we found that *FILAMENT-LIKE PLANT PROTEIN 4* (*ObFPP4-2*), four tubulin-encoding genes, *ObTUBA1*, *ObTUBA2*, *ObTUBB1-1*, and *ObTUBB1-2*, showed clear expression changes under salt stress compared with others (Figure 5; Table S1). In addition, two LOB domain-containing protein 41-encoding genes, *ObLBD41-1* and *ObLBD41-2*, which were linked to the GO term ‘response to gibberellin’, displayed downregulated expression trends after salt treatment (Figure 5; Table S1). Finally, we found that photosystem II 10 kDa polypeptide-encoding genes *ObPsbR1* and *ObPsbR2*, which were isolated from the GO term ‘photosynthesis’, were first down- and then upregulated under salt stress (Figure 5; Table S1). These genes might be directly involved in the salt stress response in sweet basil, and their functions are worthy of being studied further.

### 2.4. KEGG Pathway Analysis of Salt-Response Genes

**Figure 6:**
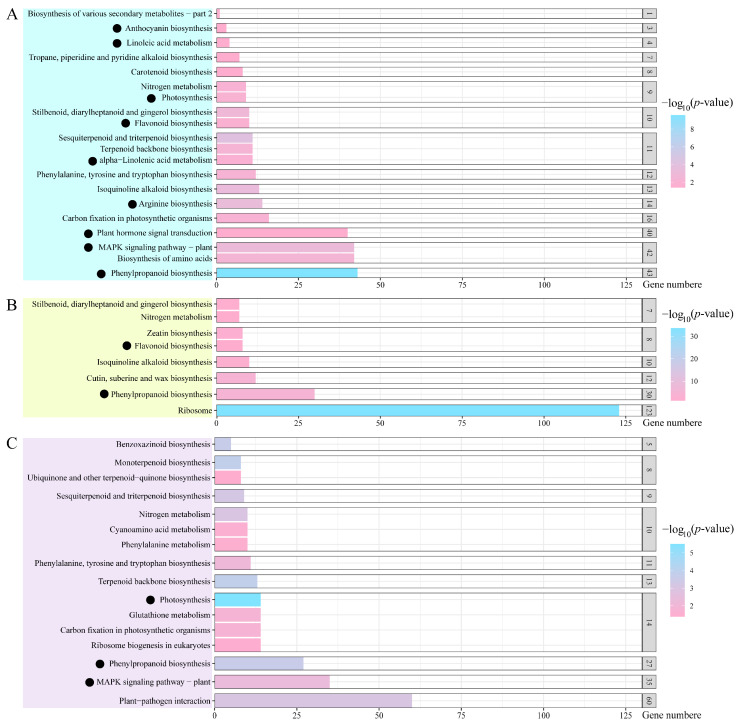
KEGG pathway analysis of DEGs in the (**A**) 6 h vs. 0 h group, (**B**) 12 h vs. 6 h group, and (**C**) 24 h vs. 12 h group. Black dots indicate the candidate KEGG pathways.

To learn more about salt-responsive DEGs, we performed a Kyoto Encyclopedia of Genes and Genomes (KEGG) analysis. Similar to the GO analysis results, we found that several DEGs from comparative groups S6 h vs. S0 h and S12 h vs. S6 h were enriched into ‘photosynthesis’ pathways (Figure 4 and Figure 6). In addition, we noticed that 40 DEGs in comparative group S6 h vs. S0 h participated in the ‘plant hormone signal transduction’ pathway, and this was partially consistent with the GO analysis data (Figure 4 and Figure 6; Table S2). We found that two auxin response factor-encoding genes, *ObARF2B* and *ObARF2A*, showed upregulated expression profiles after salt stress (Table S2), indicating that auxin might be involved in salt response regulation in sweet basil. It is well known that the mitogen-activated protein kinase (MAPK) signaling pathway takes part in regulating salt response in plants, thus, we detected 42 and 35 DEGs in comparative groups S6 h vs. S0 h and S24 h vs. S12 h, respectively (Figure 6; Table S2). Accordingly, we isolated three MAPK-encoding genes, *ObMAPK2*, *ObMAPK12*, and *ObMAPKKK20*, as candidates that might be involved in regulating the salt stress response in sweet basil (Table S2). Moreover, we found that many DEGs were isolated from metabolic substance synthetic pathways such as ‘flavonoid biosynthesis’, ‘arginine biosynthesis’, ‘phenylpropanoid biosynthesis’, and ‘α-Linolenic acid metabolism’ (Figure 6; Table S2). Among these pathways, only the ‘phenylpropanoid biosynthesis’ pathway appeared in all three comparative groups (Figure 6), indicating that phenylpropanoid metabolism might play an important role in the salt stress response in sweet basil.

### 2.5. Ectopic Expression of ObPAL2 in Arabidopsis Improved Salt Tolerance

**Figure 7:**
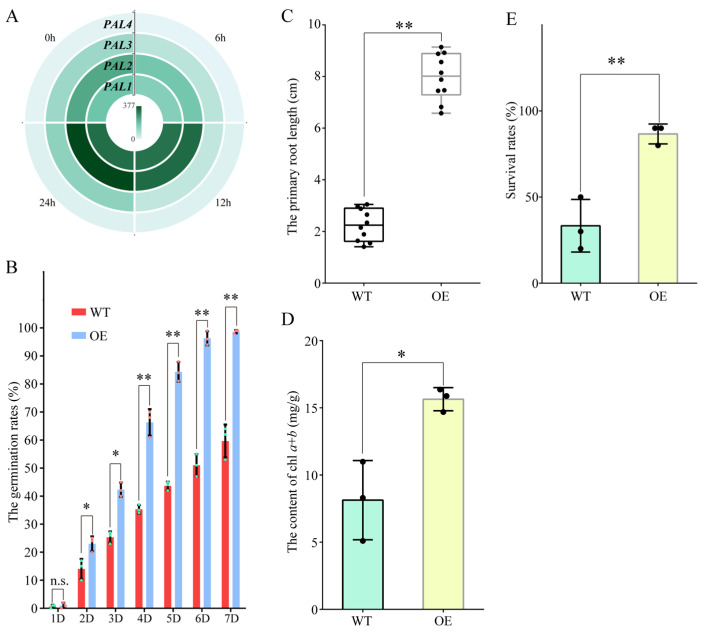
Overexpression of *ObPAL2* enhanced salt tolerance of transgenic *Arabidopsis*. (**A**) Heatmap of the expression profile of *ObPAL2* under salt conditions. (**B**) The seed germination rate statistics of the WT and transgenic *Arabidopsis* (OE) lines. *: *p* < 0.05. **: *p* < 0.01. (**C**) The primary root length statistics of the WT and transgenic *Arabidopsis* (OE) lines. **: *p* < 0.01. *n* = 10. (**D**) The chlorophyll *a* + *b* content statistics of the WT and transgenic *Arabidopsis* (OE) lines. *: *p* < 0.05. (**E**) The survival rate statistics of the WT and transgenic *Arabidopsis* (OE) lines. **: *p* < 0.01.

Due to the KEGG pathway analysis, ‘phenylpropanoid biosynthesis’ was screened out in all three comparative groups (Figure 6; Table S2); thus, we first checked the expression profiles (FPKM values) of the phenylalanine ammonia-lyase-encoding gene (PAL), which is the rate-limiting factor in the general phenylpropanoid pathway under salt stress. Four *ObPALs* were isolated, and *ObPAL2* displayed more obvious changes at four time points after the salt treatments (Figure 7A). To clarify the possible functions of *ObPAL2*, we ectopically overexpressed (OE) it in the *Arabidopsis*. Under the salt treatment, the seed germination rates in the *ObPAL2*-OE line were over 3-fold higher than the wild-type (WT) every day (except the first day) within one week (Figure 7B). In 10-day-old seedlings, the *ObPAL2*-OE line showed significantly longer primary root lengths and higher chlorophyll *a* + *b* contents than those of the WT (Figure 7C,D). Moreover, the *ObPAL2*-OE seedlings had a higher survival rate than WT after two weeks of salt treatment (Figure 7E). These results indicate that the overexpression of *ObPAL2* improved the salt tolerance of transgenic *Arabidopsis*.

### 2.6. Analysis of Transcription Factors Involved in Salt Response

**Figure 8:**
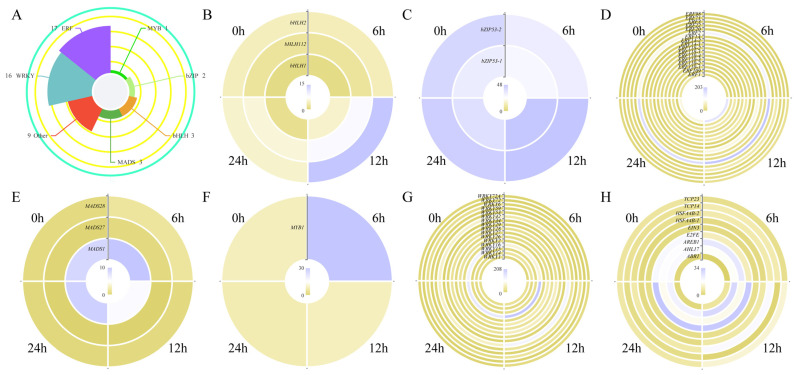
(**A**) The numbers of different types of TFs. (**B**) Heatmap of *bHLHs*. (**C**) Heatmap of *bZIPs*. (**D**) Heatmap of *ERFs*. (**E**) Heatmap of *MADSs*. (**F**) Heatmap of *MYB*. (**G**) Heatmap of *WRKYs*. (**H**) Heatmap of other TF-encoding genes. FPKM values were used.

We noticed that several DEGs were enriched in the GO term, ‘DNA-binding transcription factor activity’ (Figure 4A), and this result was a reminder that transcription factors (TFs) could play essential roles in regulating the salt response in sweet basil. A total of 51 TFs, including one MYB, two bZIPs, three bHLHs, three MADSs, seventeen ERFs, sixteen WRKYs, and nine other types of TFs, were screened out (Figure 8A). We then compared the expression patterns of each type of TF in four salt-treated samples. *ObbHLH2*, *ObbHLH12*, *ObbZIP53*-*1*, *ObERF2*, *ObMADS1*, *ObMYB1*, *ObWRKY16*, *ObE2FE*, *ObAREB1*, and *ObAHL17* showed clear expression changes during salt treatment (Figure 8; Table S3), indicating that these TFs might be involved in the salt response in sweet basil.

### 2.7. Overexpression of ObWRKY16 Enhanced Salt Tolerance in Transgenic Arabidopsis

**Figure 9:**
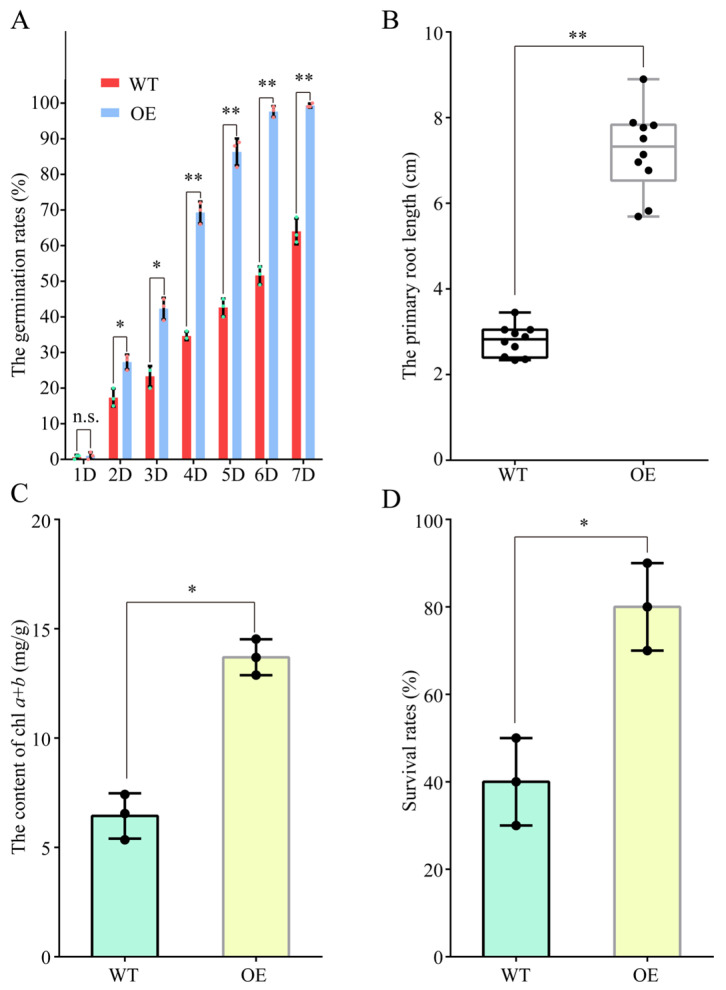
Overexpression of *ObWRKY16* enhanced salt tolerance of transgenic *Arabidopsis*. (**A**) The seed germination rate statistics of the WT and transgenic *Arabidopsis* (OE) lines. *: *p* < 0.05. **: *p* < 0.01. (**B**) The primary root length statistics of the WT and transgenic *Arabidopsis* (OE) lines. **: *p* < 0.01. n = 10. (**C**) The chlorophyll *a* + *b* content statistics of the WT and transgenic *Arabidopsis* (OE) lines. *: *p* < 0.05. (**D**) The survival rate statistics of the WT and transgenic *Arabidopsis* (OE) lines. *: *p* < 0.05.

It is well known that WRKY TFs commonly take part in regulating salt responses in plants. The fact that the expression level of *ObWRKY16* first decreased and then increased after salt treatment (Figure 8G; Table S3) suggests that this gene might be involved in regulating the salt response in sweet basil. Thus, we overexpressed *ObWRKY16* in *Arabidopsis* ectopically. As shown in Figure 9A, the *ObWRKY16*-OE line had a significantly higher seed germination rate than the WT from 2 days to 7 days under salt stress. In addition, the primary root of the OE line grew obviously longer than the WT (Figure 9B). Moreover, the OE line had a significantly higher chlorophyll *a* + *b* content than the WT (Figure 9C), demonstrating that the OE seedlings grew better than the WT. For the long-term (2 weeks) salt stress, the OE seedlings showed a significantly higher survival rate than the WT (Figure 9D). Together, the results reveal that the overexpression of *ObWRKY16* enhanced the salt tolerance of transgenic *Arabidopsis*, and this gene might be a good candidate for improving the salt resistance of sweet basil.

### 2.8. Gene Validation Using Real-Time Quantitative PCR

**Figure 10:**
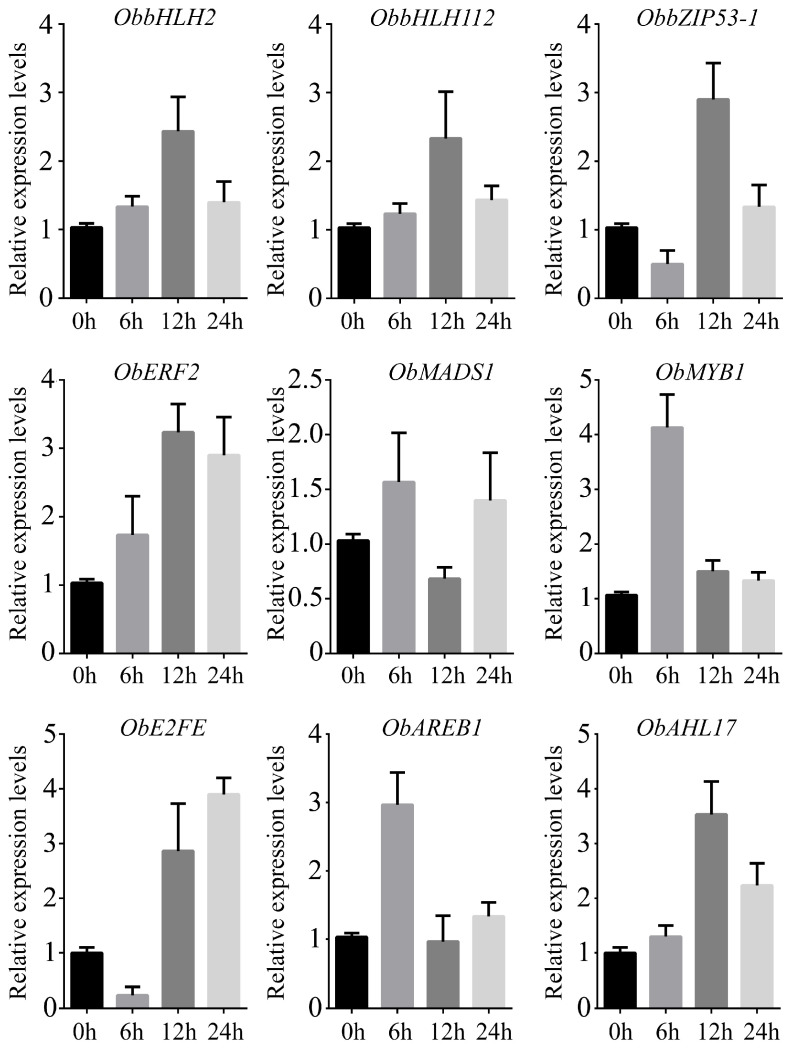
Validation of selected DEGs. qRT-PCR assays were performed.

In order to verify the reliability of the transcriptome data, qRT-PCRs were carried out. We monitored the relative expression levels of *ObbHLH2*, *ObbHLH12*, *ObBZIP53*-*1*, *ObERF2*, *ObMADS1*, *ObMYB1*, *ObE2FE*, *ObAREB1*, and *ObAHL17* in the salt-treated samples. As shown in Figure 10, all nine genes could be detected in all the salt-treated samples. The expression trend in each selected gene was similar to the transcriptome sequencing data (Figure 8 and Figure 10). These results demonstrate that our transcriptome data was highly reliable.

## 3. Discussion

As a type of aromatic plant, sweet basil can be used as both medicine and food. In some regions of Asia, sweet basil leaves are usually used as flavorful additives [12]. In addition, sweet basil contains several chemical compounds, such as terpenes, tannins, flavonoids, and phenylpropanoids, and thus is commonly utilized in traditional Chinese medicine in China. Moreover, sweet basil has racemose inflorescence and produces many flowers during the flowering phase; thus, it has ornamental value as well. So far, both the descriptive and experimental studies on salt stress responses in sweet basil are limited, although salt effects have restricted its culture and extension in many areas around the world. In this study, we observed phenotypes of sweet basil under salt stress and further confirmed sweet basil is a kind of glycophytic plant, with its seed germination rate and seedling growth obviously inhibited under salt treatment (Figure 1). Given the important values of sweet basil, it is necessary to isolate genetic resources for molecular breeding. By using comparative transcriptome analysis, several gene modules were screened out in the present work.

### 3.1. Phytohormone Signaling Pathways Might Play Important Roles in Salt Stress Response in Sweet Basil

The roles of phytohormones in regulating salt responses have been largely uncovered in recent decades. Many studies have confirmed that the gibberellin (GA) signaling pathway is involved in salt responses in plants [13]. In this study, we found that five DEGs were related to GA signaling under salt stress (Figure 5 and Table S1). Zinc finger proteins (ZFPs) can regulate the GA biosynthesis, and therefore take part in the GA signaling pathway [14,15]. An ObZFP5 gene, which can respond to salt stress in sweet basil, was screened out as a candidate for further study. For the biosynthesis of GA, the final step is catalyzed by 2-oxoglutarate-dependent dioxygenases [16]. We found that 2-oxoglutarate-dependent dioxygenase-encoding gene ObAOP1.2 was upregulated under salt treatment in sweet basil (Figure 5 and Table S1), suggesting that this gene might directly regulate GA content during salt stress in sweet basil. As members of cysteine-rich antimicrobial peptide families, gibberellin-regulated proteins (GRPs)/GASA are conserved in many plants [17]. We also isolated a GASA protein-encoding gene, ObGASA4, from the GO analysis results (Figure 4 and Figure 5, and Table S1), indicating that GA signaling might be directly involved in the salt response in sweet basil. In addition, we monitored two copies of lateral organ boundaries (LOB) domain-containing protein 41-encoding genes ObLBD41-1 and ObLBD41-2, and both of them were downregulated after salt stress (Figure 5 and Table S1). Although there were no certain connections between LBDs and salt stress regulation, it is still worth studying their functions in the sweet basil salt response in the future.

JA is a stress-related hormone involved in salt stress responses in many plants [1]. Usually, JA contents are increased, and then JA signaling is activated during salt stress [18,19,20]. The TIFY (TIF[F/Y]XG) gene family is a plant-specific gene family in which many members take part in the JA signaling pathway [21]. Several works have shown that TIFY genes contribute to salt stress tolerance. TdTIFY11a, which was cloned from Triticum Durum, was specifically induced after salt treatment. Transgenic Arabidopsis lines overexpressing TdTIFY11a displayed increased germination and growth rates after high salinity treatment [22]. In soybeans, overexpression of GmTIFY10e and GmTIFY10g enhanced salt tolerance, while the RNAi of GmTIFY10e and GmTIFY10g significantly affected plants’ sensitivity to salt stress [23]. In the present study, we isolated four ObTIFY genes and found that ObTIFY6B and ObTIFY10A were induced after salt treatment in sweet basil (Figure 5 and Table S1), indicating that these genes might play roles in regulating the salt response in sweet basil.

It is well understood that auxin plays key roles in several aspects of growth and development and stress responses in plants [24,25,26,27]. Several studies have indicated that auxin levels are altered under salt stress, and the processes regulating auxin biosynthesis under salt stress are still largely unknown. As a phytohormone, auxin can regulate gene expression via the functionally distinct transcription factors ARFs [28]. ARFs have been revealed to play roles in salt stress responses in plants. For instance, the knockdown of SlARF2 confers enhanced tolerance to salt stresses in tomatoes (*Solanum lycopersicum* L.) [29]. A gene named IbARF5, which was cloned from the sweet potato, increased the contents of carotenoids and enhanced the tolerance to salt and drought in transgenic Arabidopsis [30]. In this study, we isolated two ARFs, ObARF2B and ObARF2A, from sweet basil, and their expression levels were induced under salt stress (Table S2), indicating that the auxin-mediated signaling pathway took part in the salt stress response in sweet basil.

### 3.2. Photosynthesis Was Inhibited in Sweet Basil under Salt Treatment

Salt stress can damage the photopigment system and restrain chlorophyll production, severely inhibiting photosynthesis [31]. Usually, salt stress seriously reduces nitrogen and magnesium ion uptake, inhibiting chlorophyll biosynthesis. In Acacia auriculiformis, the contents of chlorophyll a, chlorophyll b, and total chlorophyll are all reduced under seawater-induced salt stress [32]. In this study, we also measured the contents of chlorophyll a + b in sweet basil, and the results show that the level of chlorophyll a + b was decreased after salt stress (Figure 1I), indicating that salt stress influenced the photosystem by damaging photosynthetic pigments in sweet basil.

The photosystem II (PSII) complex is located in the thylakoid membrane of higher plants, algae, and cyanobacteria, and drives the water oxidation process of photosynthesis [33], thus PSII is important for light energy conversion and photosynthetic efficiency. However, salinity stress can significantly inhibit photosystem II [34]. PsbR is a low molecular mass protein in PSII and is found only in the nucleus of green algae and higher plants [33]. The mature PsbR protein, previously named the 10-kDa polypeptide, has 99 amino acids with 10.3 kDa molecular mass in most species [33]. Herein, we found that two PsbR protein-encoding genes, ObPsbR1 and ObPsbR2, demonstrated expression alterations after salt stress in sweet basil (Figure 5; Table S1), indicating that PSII is influenced by salt treatment. Taken together, our data suggest that photosynthesis is inhibited in sweet basil under salt treatment.

### 3.3. Transcription Factors Might Play Important Roles in the Salt Stress Response in Sweet Basil

Transcription factors (TFs) are widely involved in regulating plants’ response to abiotic and biotic stresses, especially salt stress. Although over 80 types of TFs exist in plants, only a few types of TFs, such as basic leucine zipper (bZIP), Apetala (AP2), NAC (NAM, ATAF1,2, CUC2), basic helix–loop–helix (bHLH), myeloblastosis (MYB), WRKY, and ABA-binding factor (ABF), have been revealed to have regulatory roles in salt responses [35]. bZIP transcription factors in plants can interact with several kinds of elements, including ABRE(CCACGTGG) elements, PB(TGAAAA) elements, GLM (GTGAGTCAT) elements, and so on [36]. A large number of bZIP TFs are associated with salt stress in plants. In Arabidopsis, AtbZIP1, AtbZIP17, and AtbZIP28 were involved in regulating salt resistance [36]. In this study, we screened out one bZIP TF-encoding gene, ObbZIP53-1, with an increased expression profile under salt stress (Figure 8; Table S3), indicating that this gene might play a role in regulating the salt response in sweet basil. The bHLH family is the second largest family in plants. bHLH TFs regulate the expression of abundant genes involved in a wide range of regulatory pathways [37]. Many studies have reported that bHLH TFs take part in plants’ response to salt stress. In sorghum, overexpression of SbbHLH85 resulted in a significantly increased number and length of root hairs through ABA and auxin signaling pathways, increasing the absorption of Na^+^. Thus, SbbHLH85 is a negative regulator in the salt tolerance of sorghum [38]. In pepper, the expression of CabHLH035 increased after salt treatment, and the transient expression of this gene enhanced its salt tolerance. In addition, the ectopic expression of CabHLH035 in Arabidopsis increased the salt stress tolerance, whereas knocking down the expression of CabHLH035 in pepper plants resulted in decreased salt tolerance [39]. We also isolated two bHLH TFs-encoding genes, ObbHLH2 and ObbHLH12, and their expression levels increased following salt treatment (Figure 8; Table S3), suggesting that these genes might have functions in the salt response in sweet basil. APETALA2/ethylene responsive factor (AP2/ERF) is involved in plant responses to environmental stresses including salt stress [40]. Several studies report that stress-responsive AP2//ERF genes regulate the tolerance of plants to stress. In soybeans, overexpression of GmERF3 enhanced tolerance to salt and drought stresses in transgenic tobacco [41]. In Arabidopsis, overexpression of AtERF1 significantly improved tolerance to salt and drought stresses [42]. In the present study, we found that ObERF2 was induced under salt treatment in sweet basil (Figure 8; Table S3), indicating that the ethylene signaling pathway participated in salt stress regulation, and this gene might be a candidate for changing the salt resistance of sweet basil. As one of the most widespread transcription factor families, MYB TFs participate in plants’ response to stresses by binding with MYB cis-elements in promoters of target genes [43]. In Arabidopsis, MYB42 positively modulates salt tolerance through the regulation of SALT OVERLY SENSITIVE 2 (SOS2) expression [44]. Moreover, MYB74 plays a positive role in the salt response as well. The transgenic plants overexpressing MYB74 showed hypersensitivity to salt treatment during seed germination [45]. We also obtained an MYB TF-encoding gene, ObMYB1, from sweet basil. Its expression was induced by salt treatment (Figure 8; Table S3). This gene might play a role in salt response regulation in sweet basil.

It is well known that WRKY TFs constitute one of the largest TF families in plants and have crucial functions in plant growth and development, defense regulation, and stress responses [46]. Ever-increasing numbers of studies, which mainly uncover salt stress regulation, indicate WRYK TFs are key regulators in the network of salt resistance in plants. Overexpression in rice of the isolated salt-responsive ZmWRKY114 gene from maize resulted in decreases in both salt-stress tolerance and abscisic acid sensitivity by regulating stress- and abscisic acid-related gene expression [47]. In peanuts, the expression AhWRKY75 was induced by NaCl stress treatment. After salt treatment, the AhWRKY75-overexpressing peanuts grew better than WT plants [48]. In this study, we also identified one WRKY TF-encoding gene, ObWRKY16, from sweet basil. Its expression levels were upregulated after salt treatment (Figure 8; Table S3). In order to understand more, we generated an ObWRKY16-overexpressing Arabidopsis line. Under salt treatment, the transgenic line showed higher seed germination rates, primary root lengths, chlorophyll *a*+*b* contents, and survival rates than those of the WT (Figure 9). These results indicate that ObWRKY16 might be a positive regulator in salt stress response regulation and could be a good candidate for promoting salt tolerance in sweet basil. Taken together, TFs play essential roles in regulating the salt stress response in sweet basil.

### 3.4. Cytoskeleton Structure Might Be Damaged during Salt Stress in Sweet Basil

A plant cytoskeleton is composed of actin filaments (F-actin) and microtubules (MTs), and constantly undergoes dynamic alteration in architecture. Many studies have shown that the cytoskeleton plays a crucial role in a wide variety of cellular processes such as cell shape determination, cell movement, tip growth, vesicle trafficking, and responses to stress stimuli [49]. During salt stress, the cytoskeleton can help plants resist stress conditions through dynamic organizational changes [1]. Salt stress triggers changes in the cytoskeleton architecture by modulating nucleation and polymerization, severing and depolymerizing, crosslinking/bundling, and growth/shrinkage [50]. Consistent with this, we found that several DEGs were enriched in cytoskeleton-related GO terms, such as ‘microtubule polymerization or depolymerization’, ‘structural constituent of cytoskeleton’, and ‘microtubule-based process’ (Figure 5; Table S1), indicating that the dynamics of the cytoskeleton in sweet basil might be changed after salt treatment. In addition, we isolated several candidates, including ObTUBA1, ObTUBA2, ObTUBB1-1, and ObTUBB1-2, which were key parts of the microtubule, by GO analysis. These genes might be good genetic resources for molecular breeding of sweet basil.

### 3.5. Metabolite Response to Salt Stress in Sweet Basil

Plants can biosynthesize specialized metabolites to adapt to environmental stresses such as biotic and abiotic stresses [51]. Various plant metabolites, such as proline, flavonoids, terpene, phenylpropanoid, α-linolenic acid, and so on, have a protective role during abiotic stress, especially salt stress. As a kind of amino acid, proline has long been known to act as a compatible osmolyte to counteract drought and salt stresses [52]. Thus, proline metabolism plays an essential role in the plant’s response to salt stress conditions. In Arabidopsis, a bHLH TF, MYC2, imparts salt intolerance by regulating proline biosynthesis [53]. In this study, we found a bHLH TF-encoding gene, ObbHLH112-2, and its expression was regulated by salt treatment in sweet basil (Figure 5; Table S1), indicating that proline metabolism was involved in the salt stress response in sweet basil. Flavonoids act as free radical scavengers and play crucial roles in resisting abiotic stress [54]. We found that many DEGs were clustered within the ‘flavonoid biosynthesis’ pathway during salt stress in sweet basil (Figure 6), demonstrating that the flavonoid biosynthesis pathway was activated. Several studies have shown that environmental stresses, such as drought, cold, salt, and heat, can induce changes in fatty acid composition, especially the content of linolenic acid [55]. In our data, α-linolenic acid and linolenic acid metabolism pathways were screened out (Figure 6), suggesting that fatty acid might be involved in the salt stress response in sweet basil, and the functions of the enriched DEGs (Table S2) should be studied further. In addition, we also found that ‘anthocyanin biosynthesis’ and ‘phenylpropanoid biosynthesis’ pathways were displayed after salt stress (Figure 6). Phenylpropanoids contribute to almost all aspects of plant responses to biotic and abiotic stresses [56]. Anthocyanins are water-soluble pigments, which are synthesized by the flavonoid metabolic pathway [57]. Anthocyanins are first synthesized in the cytosol, and then modified to various anthocyanin derivatives and transported to vacuoles. The main player in anthocyanin biosynthesis is the phenylpropanoid pathway. In this study, we found the KEGG pathway ‘phenylpropanoid biosynthesis’ was isolated in all three comparative groups (Figure 6), indicating that this pathway might play an important role in the salt stress response in sweet basil. Given this, we cloned a salt-induced gene ObPAL2 and overexpressed it in Arabidopsis. The data displayed that overexpression of ObPAL2 enhanced salt tolerance in transgenic Arabidopsis (Figure 9). Terpenoids represent the largest and most diverse class of chemicals among the myriad compounds produced by plants. Plants employ terpenoid metabolites for a variety of protection types in the abiotic and biotic environment [58]. We noticed that 9 ObTPS genes were enriched in the GO term ‘terpene synthase activity’, and ObTPS6 and ObTPS7 were upregulated after salt treatment (Figure 5; Table S1), suggesting that terpene might play essential roles in the salt stress responses in sweet basil. In conclusion, metabolites might be involved in the salt stress response in sweet basil due to the fact that their biosynthesis pathways are activated during salt treatment.

## 4. Materials and Methods

### 4.1. Plant Materials and Growth Conditions

Sweet basil seeds and Arabidopsis seeds (Col-0) were kept in our laboratory. Both sweet basil and Arabidopsis seeds and seedlings were cultivated in a light incubator. For experiments with the sweet basil, the growth conditions were 25 °C and a 16 h light/8 h dark photoperiod. For the Arabidopsis, the growth conditions were 22 °C and a 16 h light/8 h dark photoperiod.

### 4.2. Measurement of Growth and Physiological Indexes

For the seed germination test of sweet basil, a total of 50 seeds were sown in dishes with filter papers soaked with water or 150 mmol/L of NaCl solution. For the seed germination test with Arabidopsis, 100 seeds were sown on half-strength Murashige and Skoog (MS) medium with or without 150 mmol/L of NaCl (final concentration). The data on seed germination was continuously recorded for 7 days. The primary root length of the 10-day-old sweet basil and Arabidopsis seedlings was measured using ImageJ software (https://fiji.sc/; accessed on 3 March 2018). The chlorophyll content of the leaf tissues was measured, according to the method described previously [59]. The fresh weight of the seedlings was measured using 10 seedlings. For the salt tolerance assay, 4-week-old plants of WT and transgenic Arabidopsis lines grown in a soil mixture were watered with 150 mmol/L of NaCl continuously for 2 weeks. The survival rates were then calculated [60].

### 4.3. Sample Preparation for Transcriptome Sequencing

The 14-day-old sweet basil seedlings were treated with 150 mmol/L of NaCl solution (experimental group) or water (control), and the seedlings were collected at 0 (before treatment), 6, 12, and 24 h post-treatment. All the samples were quick-frozen using liquid nitrogen and stored at −80 °C.

### 4.4. RNA-Seq Data Analysis

The RNeasy Plant Mini Kit (QIAGEN, Dusseldorf, Germany) was used to extract total RNA. The RNase-Free DNase Set (QIAGEN, Germany) was used to eliminate the contamination of genomic DNA in the RNA samples. The RNA quality was monitored using a micro-spectrophotometer (NanoDrop 2000c, ThermoFisher, Waltham, MA, USA). The methods of the RNA-seq data analysis were clearly described previously [61]. Genes with an adjusted *p*-value < 0.05 found by DESeq were assigned as differentially expressed. Genes with a fold change ≥ 1 and false discovery rate < 0.05 were considered as DEGs. The fragments per kilobase of transcript per million mapped reads method was used to calculate the expression abundances of the genes. The GO enrichment analysis of DEGs was implemented by the GOseq R packages based on Wallenius noncentral hypergeometric distribution. The KEGG pathway analysis was performed (*p* ≤ 0.05) using BlastX searches against the KEGG pathway database (https://www.genome.jp/kegg/pathway.html accessed on 20 April 2024).

### 4.5. Validation of DEGs by qRT-PCR

Reverse-transcription PCR was performed using PrimeScript^T^ RT Master Mix (Takara, Japan). qRT-PCR was applied as described previously [61]. Each experiment was repeated three times. The relative expression level of each gene was calculated using the 2^−Δ Δ Ct^ method [62]. The sweet basil 18s ribosomal RNA-encoding gene was used as the inner reference [63]. The primers are listed in Table S4.

### 4.6. Vector Construction and Transgene

The coding sequence of ObWRKY16 and ObPAL2 (no stop codon) were inserted into the pENTR/D-TOPO vector. Then, the gateway reaction was applied to pB7WG2 containing CaMV 35S promoter fused with GFP to generate ObWRKY16-pB7WG2 and ObPAL2-pB7WG2. The Agrobacterium-mediated transformation method was performed to generate the transgenic Arabidopsis lines [64]. The primers are listed in Table S4.

### 4.7. Statistical Analysis and Data Availability

One-way ANOVA followed by the Tukey–Kramer test (*p* < 0.01) or Student’s *t*-test (two-tailed, *p* < 0.05) was performed using SPSS software (v. 22) to detect differences as required. The RNA-seq data sets were submitted to the National Genomics Data Center (https://ngdc.cncb.ac.cn/ accessed on 20 April 2024) with the accession number CRA016103.

## 5. Conclusions

Due to its high nutritional and officinal value, sweet basil is worth widely planting and extending. However, soil salinity severely limits the extension of sweet basil because it is sensitive to salt stress. Our phenotypical data illustrated this point as well. In this study, we performed a transcriptome analysis to uncover the gene modules involved in the salt stress response in sweet basil. By setting out the comparative groups, we gained a number of DEGs that respond to salt stress. GO and KEGG enrichment analysis showed that TFs, phytohormone signaling, the photosynthetic system, and metabolite biosynthesis were the main modules that might be involved in the salt stress response in sweet basil. More importantly, we generated *ObWRKY16* and *ObPAL2* overexpressing *Arabidopsis* lines and found that these genes contribute to salt tolerance in transgenic *Arabidopsis*, suggesting that these genes might directly take part in salt resistance in sweet basil as positive regulators. In a word, our results provide the transcriptome data on the salt stress response in sweet basil. Meanwhile, we screened out several candidates, such as the above-mentioned *ObWRKY16* and *ObPAL2*, for the molecular breeding of sweet basil.

## Data Availability

The data presented in this study are available upon request.

## Supplementary Materials

The following supporting information can be downloaded at: https://www.mdpi.com/article/10.3390/plants13111487/s1, Table S1: Information on DEGs enriched in the representative GO terms; Table S2: Information on DEGs enriched in the representative KEGG pathways; Table S3: Information on TFs in this study; Table S4: The primers used in this study.
